# Genetic Landscape of Factor VII Deficiency: Insights from a Comprehensive Analysis of Pathogenic Variants and Their Impact on Coagulation Activity

**DOI:** 10.3390/ijms25042384

**Published:** 2024-02-17

**Authors:** Barbara Preisler, Behnaz Pezeshkpoor, Anja Merzenich, Sandra Ohlenforst, Heiko Rühl, Vytautas Ivaškevičius, Ute Scholz, Hagen Bönigk, Wolfgang Eberl, Barbara Zieger, Anna Pavlova, Johannes Oldenburg

**Affiliations:** 1 Institute of Experimental Hematology and Transfusion Medicine, University of Bonn, Faculty of Medicine, University Clinic Bonn, 53127 Bonn, Germany; barbara.preisler@ukbonn.de (B.P.); behnaz.pezeshkpoor@ukbonn.de (B.P.); sandra.ohlenforst@ukbonn.de (S.O.); heiko.ruehl@ukbonn.de (H.R.); vytautas.ivaskevicius@ukbonn.de (V.I.); anna.pavlova@ukbonn.de (A.P.); 2 Center of Hemostasis, MVZ Labor Leipzig, 04289 Leipzig, Germany; u.scholz@labor-leipzig.de; 3 MVZ Limbach Magdeburg, Lab Dr. Franke, Bönigk and Colleagues, Center of Coagulation Disorders and Vascular Diseases, 39104 Magdeburg, Germany; dr.boenigk@gerinnungszentrum-md.de; 4 Pediatric Hematology and Oncology, Klinikum Braunschweig, 38118 Braunschweig, Germany; w.eberl@klinikum-braunschweig.de; 5 Department of Pediatrics and Adolescent Medicine, Division of Pediatric Hematology and Oncology, Medical Center–University of Freiburg, Faculty of Medicine, 79110 Freiburg, Germany; barbara.zieger@uniklinik-freiburg.de

**Keywords:** coagulation factor VII, factor VII deficiency, genetic testing, hemostasis, high-throughput sequencing

## Abstract

Congenital factor VII (FVII) deficiency is a rare genetic bleeding disorder characterized by deficient or reduced activity of coagulation FVII. It is caused by genetic variants in the *F7* gene. We aimed to evaluate the rate of detection of pathogenic variants in the *F7* gene in a large group of patients with FVII deficiency and investigate the correlations between the *F7* genotype and FVII activity (FVII:C). Moreover, the influence of the common genetic variant rs6046: c.1238G>A; p.(Arg413Gln), designated as the M2 allele, on FVII:C was investigated. Genetic analysis of the *F7* gene was performed on 704 index patients (IPs) using either direct Sanger- or next-generation sequencing. Genetic variants were detected in 390 IPs, yielding a variant detection rate (VDR) of 55%. Notably, the VDR exhibited a linear decline with increasing FVII:C levels. We identified 124 genetic variants, of which 48 were not previously reported. Overall, the frequency of the M2 allele was considerably higher in patients with mild deficiency (FVII:C > 20 IU/dl). Furthermore, IPs lacking an identified pathogenic variant exhibited a significantly higher prevalence of the M2 allele (69%) compared to IPs with a disease-causing variant (47%). These results strongly support the association of the M2 allele with decreased FVII:C levels. This study shows the utility of FVII:C as a predictive marker for identifying pathogenic variants in patients with FVII deficiency. The M2 allele contributes to the reduction of FVII:C levels, particularly in cases of mild deficiency.

## 1. Introduction

Factor VII (FVII) is a zymogen of a vitamin K-dependent serine protease that is synthesized in the liver and plays an important role in the coagulation network [1]. FVII deficiency is a rare bleeding disorder characterized by deficient or reduced FVII activity (FVII:C). An inherited FVII deficiency is the most common among the rare inherited bleeding disorders, with a prevalence of 1:300,000 to 1:500,000 [2]. Clinical presentation of FVII deficiency is highly heterogeneous, ranging from asymptomatic to severe life-threatening bleedings, often manifesting in early infancy [3].

Factor VII deficiency is caused by a heterogeneous spectrum of pathogenic genetic variants of the *F7* gene, following an autosomal recessive pattern of inheritance [4]. The *F7* gene is located at the terminus of chromosome 13 (13q34), 2.8 kilobases (kb) proximate to the *F10* gene, and therefore large rearrangements often involve both genes, leading to combined FVII and factor X (FX) deficiency [5,6]. Pathogenic variants of the *F7* gene lead to the reduction of FVII activity levels in plasma. In contrast to the majority of bleeding disorders, where disease severity often correlates with residual protein activity, FVII deficiency does not consistently follow this pattern. The variability and insensitivity of FVII:C assays may contribute to this discrepancy, alongside potential environmental and genetic factors, within or beyond the *F7* gene.

The European Association for Haemophilia and Allied Disorders (EAHAD) database (https://f7-db.eahad.org/ accessed to on 1 July 2021) [7] reports over 271 different pathogenic genetic variants within the *F7* gene. These variants include missense, nonsense, small insertion/deletion, splice site defects and large deletion, distributed throughout the entire *F7* gene and affecting all protein domains, with the majority being missense variants.

Additionally, the *F7* gene harbors several likely benign variants, which can modulate FVII levels [8,9,10], potentially simulating heterozygous FVII deficiency in a homozygous state [11]. The minor alleles of rs5742910 (−323 10 bp insertion) in the gene’s promoter region and rs6046 (c.1238G>A; p.(Arg413Gln) in exon 9) have consistently shown a strong correlation with lower levels of plasma FVII:C [11,12]. Given their capacity to lower FVII plasma levels, these variants may contribute to the severity of disease, suggesting clinical relevance in FVII-deficiency cases [13,14].

The present study aims to evaluate the variant detection rate (VDR) of pathogenic variants in the *F7* gene in a large group of patients subjected to molecular testing based on reduced FVII activity levels. Additionally, we intend to investigate the correlation between the *F7* genotype and FVII coagulant activity and explore the influence of a common genetic variant on residual FVII activity in our patient cohort compared to a control group.

## 2. Results

### 2.1. Association of Variant Detection Rate with FVII:C Levels

**Figure 1:**
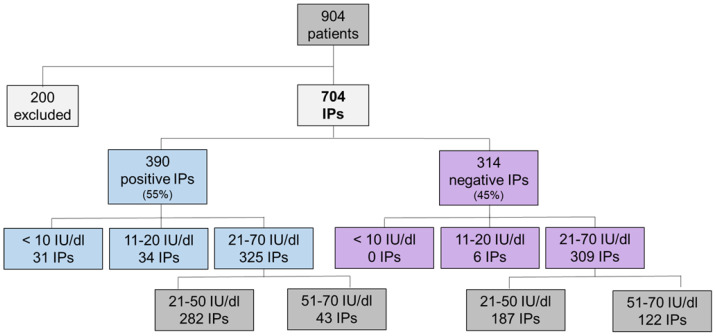
Patient cohort with FVII deficiency. Overall prevalence of patients with an identified genetic defect (positive IPs, blue colored boxes) and patients with no identified genetic defect in the *F7* gene coding sequence and exon–intron boundaries (negative IPs, purple colored boxes). IPs—index patients. Factor VII activity is given in IU/dl.

A cohort of 704 IPs with reduced FVII:C (<70 IU/dl) [15] underwent genetic analysis (Figure 1). We investigated the capacity to identify variants (pathogenic, likely pathogenic and VUS) in relation to FVII:C, termed the Variant Detection Rate (VDR). In 390 IPs, we identified a genetic variant, yielding a total VDR of 55%. The cohort was categorized into three groups: (i) severe FVII deficiency (FVII:C < 10 IU/dl), (ii) moderate FVII deficiency (FVII:C of 11–20 IU/dl) and (iii) mild FVII deficiency (FVII:C of 21–70 IU/dl).

**Figure 2:**
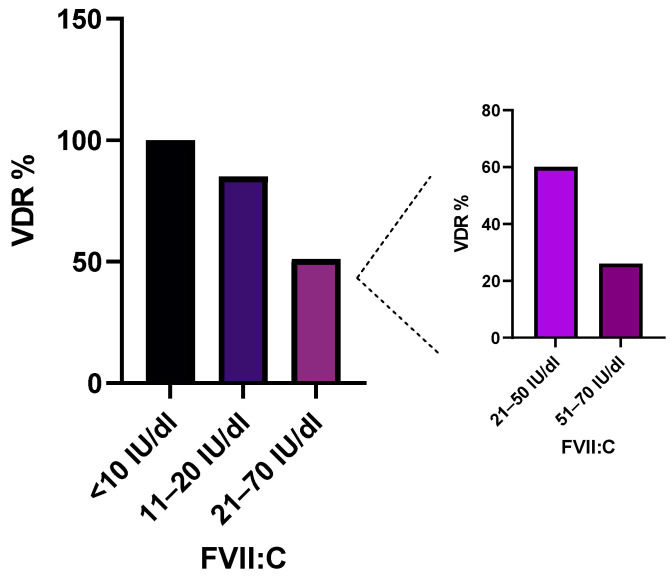
Variant detection rate (VDR) in patients with FVII deficiency.

The first group encompassed 31 IPs, where at least one pathogenic genetic variant was identified, leading to a VDR of 100% (Figure 2). These genetic variants were predominantly found in exon 9, existing in either homozygous or compound heterozygous states. The second group comprised 40 IPs with moderate FVII deficiency from which genetic variants were detected in 34 patients, leading to a VDR of 85% (Figure 2). The zygosity of the identified variants exhibited a diverse pattern, with nearly 50% in homozygous or compound heterozygous states, and the remainder displaying heterozygosity.

The last and the largest group involved 634 IPs. In approximately half (325 IPs), genetic variants were identified, defining a VDR of 51% (Figure 2). All identified variants were in heterozygous states. In the majority of patients, the genetic variant was found in exon 9, due to the high prevalence of a recurrent variant, c.1061C>T, p.Ala354Val (MAF: 5.6 × 10^−4^). Interestingly, when patients were further sub-divided into two subgroups based on FVII:C levels (21–50 IU/dl and 51–70 IU/dl), a decline in the VDR was observed, dropping from 60% to 26%, respectively. Furthermore, 87% of all detected genetic alterations were identified in the group of patients with FVII:C 21–50 IU/dl, while only 13% were identified in patients with FVII:C 51–70 IU/dl (Figure 1 and Figure 2).

### 2.2. Impact of the rs6046 Variant on FVII Activity

We explored the impact of the rs6046 variant (p.Arg413Gln, previously Arg353Gln), also known as the M1/M2 polymorphism, on FVII activity levels. The p.Arg413 and the minor allele p.Gln413 were assigned further as M1 and M2 alleles, respectively. We evaluated the frequency of M1 and M2 alleles in patient groups with identified variants excluding the patients with large deletions (381 IPs), those without genetic variants (314 IPs) and a control group (217 samples).

**Figure 3:**
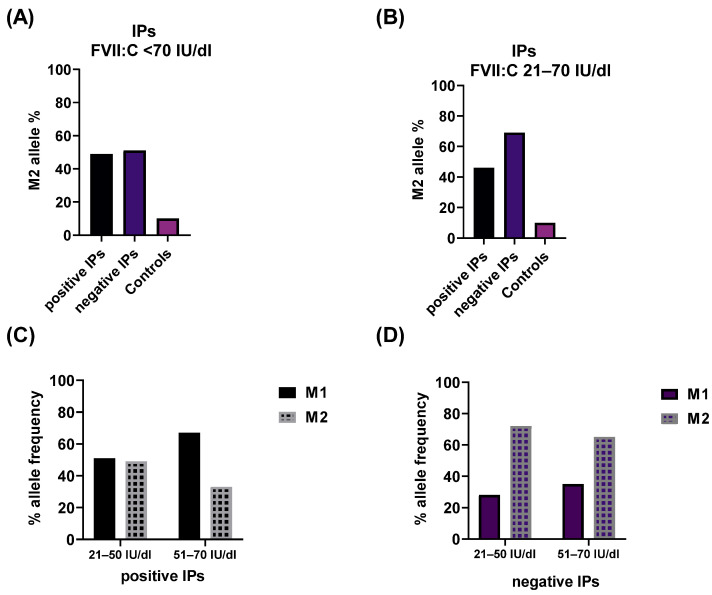
Frequency of the M2 allele: (**A**) M2 distribution in whole cohort and controls. Patients were divided based on identification of genetic lesion (positive) or not (negative); (**B**) M2 distribution in mild FVII-deficient IPs—positive—with detected genetic variant, negative—without detected genetic variant; (**C**) allele frequency of M1 and M2 alleles in mild FVII-deficient patients with detected genetic variant—group 1 FVII:C 21–50 IU/dl, group 2 FVII:C 51–70 IU/dl; (**D**) allele frequency of M1 and M2 alleles in mild FVII-deficient patients without detected genetic variant—group 1 FVII:C 21–50 IU/dl, group 2 FVII:C 51–70 IU/dl.

In the overall patient cohort, no significant difference in the frequency of the M2 allele was observed between those with and without genetic defects (49% vs. 51%) (Figure 3A). For patients with severe FVII deficiencies, the association of the M2 allele with FVII:C was challenging to establish due to the homozygous or compound heterozygous state of the identified genetic defect. Thus, the impact of the minor M2 allele was masked by the disease-causing variant.

Data from patients with moderate FVII deficiencies indicated that nearly all patients with identified disease-causing variants in homozygous or compound heterozygous states displayed an M2 allele in heterozygous combination with the M1 allele. Conversely, in 60% of IPs with only one disease-causing variant in the heterozygous state, the M2 variant was found in a homozygous state. In this group, patients without genetic defects were notably underrepresented (6 form 314), limiting conclusions about the M2 allele.

Interesting data were attained when comparing the frequency of the M2 variant in patients with mild FVII deficiencies with and without pathogenic variants to the control group. The frequency of the M2 allele was calculated as 46%, 69% and 10%, respectively (Figure 3B). The M2 allele was overrepresented in patients without genetic defects with mild FVII deficiencies compared to the entire cohort. Conversely, the M2 allele was notably underrepresented in the control group compared to the patient cohort (Figure 3A,B).

Furthermore, we explored the frequency of the M2 allele after sub-dividing the group of mildly deficient patients, as described earlier. When a genetic defect was identified, no difference was observed in the frequency of the M1 and M2 alleles when FVII:C was between 21 and 50 IU/dl. Conversely, the M1 allele was overrepresented (67%) in FVII activity levels of 51–70 IU/dl (Figure 3C). Despite the low frequency of the M1 allele in the group of patients without genetic defects, the relationship of the M1/M2 alleles in both subgroups remained similar (Figure 3D).

Finally, we compared the M2 allele frequency in the group of patients with and without genetic alterations with FVII:C 51–70 IU/dl. The data showed a twofold higher frequency of the M2 allele in patients without detected genetic variants. Patients were further sub-divided into two subgroups based on FVII:C levels (21–50 IU/dl and 51–70 IU/dl).

### 2.3. Profile of Identified Genetic Variants

Within the entire cohort, 124 distinct genetic variants with a minor allele frequency (MAF) of less than 1% (pathogenic, likely pathogenic and VUS) were identified in 390 IPs. Of these, 71 were reported in the EAHAD database as pathogenic, while 53 were assigned as VUSs. Subsequently, a portion of the latter were reclassified based on segregation family analyses and type of genetic defect (Table 1).

**Figure 4:**
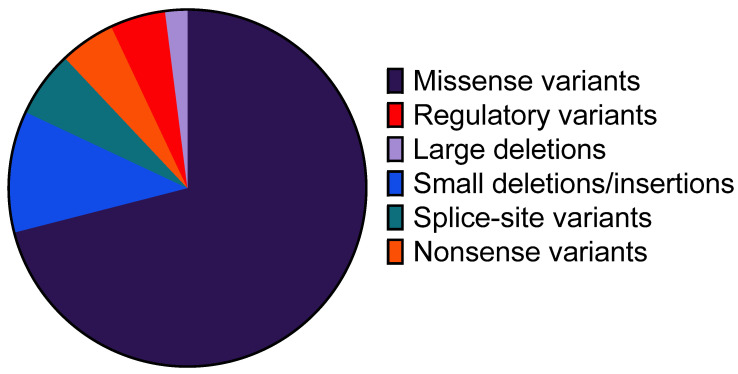
Molecular genetic profile in 704 patients with FVII deficiency.

The profile of identified defects comprised nearly all types of genetic alterations, including missense, nonsense, splice-site and regulatory variants, small deletion/insertion and large deletions, except large duplications. The most frequent types of defects were missense variants, accounting for 71% of all alterations (Figure 4). The distribution of the remaining defects was as follows: small deletions/insertions (11%), splice-site variants (6%), nonsense variants (5%), regulatory variants (5%) and large deletions (2%). In terms of localization, the majority of the detected variants were located in exon 9, the largest segment of the *F7* gene. However, genetic alterations were observed in all other exons.

### 2.4. Genetic Variants Detected in Multiple IPs

Forty-five variants were detected in more than one IP, encompassing 85% of our cohort (331 IPs). The identified genetic defects within this large group of patients included all types of genetic alterations (missense, nonsense, splice-site, small deletions/insertion and large deletions). The IPs with variants were further categorized into three sub-groups based on the incidence of the same genetic variant: genetic variants detected in (i) 2 to 5 IPs, (ii) 6 to 9 IPs and (iii) more than 10 IPs (Supplementary Table S1). Among these, four variants (p.Ala304Val, p.Gly157Ser, p.Val312Met and p.Ala354Val) in the last group were recurrent and found in a large number of patients. Notably, the variant p.Ala354Val was detected in 105 IPs, either alone or linked to a small deletion (p.Pro464Hisfs) in the same exon. The majority of the identified variants were reported in the databases as pathogenic and only five were classified as VUSs. Two variants were reclassified as likely pathogenic due to their type and family segregation analyses. Three alterations remained with an uncertain pathogenicity. A constellation of two missense variants resulting from three substitutions in exon 3 was inherited simultaneously in 10 IPs (p.Cys82Phe(;)Glu86Val). Moreover, this group included 10 IPs with large deletions, with the most prevalent being a complete deletion of the *F7* gene (observed in 5 IPs). In six IPs, the deletion of the *F7* gene was combined with complete or partial deletion of the F10 gene.

### 2.5. Genetic Variants Detected in a Single IP

The remaining 79 genetic variants were considered unique as they were exclusively detected in a single IP, either alone or in combination with another defect in the *F7* gene. Thirty-one variants were classified as pathogenic based on database reports, while the remaining variants were defined as VUSs (Table 1). Further data evaluation led to the reclassification of several variants as likely pathogenic due to the type of genetic alteration (insertion/deletion, consensus splice-site affecting position +/−3, nonsense) or family segregation analyses.

## 3. Discussion

In this study, we evaluated the association of the molecular profile and FVII:C in a large cohort of 704 unrelated FVII deficiency patients. The genotype–phenotype relationship in FVII deficiency has already been analyzed in several smaller cohorts showing the high clinical, laboratory and genetic variability of the disease [9,16,17]. Our findings reinforce the inconsistent relationship between the *F7* genotype and residual FVII coagulant activity, shedding light on this complex interplay within a large, well-characterized genetic cohort. We categorized our patient cohort into three groups based on FVII:C levels [17,18,19], as recommended by Peyvandi et al.: severe deficiency (FVII:C < 10 IU/dl), moderate deficiency (FVII:C 11–20 IU/dl) and mild deficiency (FVII:C > 20 IU/dl) [20,21,22].

The profile of pathogenic variants in our cohort is in concordance with the findings of other studies, with missense variants being the most prevalent (71%) [7,17]. The majority of pathogenic variants are located in exon 9. Whether this is due to the size of the exon (the largest) or a higher variant rate can be speculated [7]. Additionally, p.(Ala354Val), identified in 105 Ips, is also located in this exon.

It is important to indicate that in the majority of cases large deletions in the *F7* gene were coupled with a partial or complete deletion of the *F10* gene, often due to a larger deletion involving a bigger part of the long arm of chromosome 13 [5]. This raises the question of whether, in patients with large identified deletions in the *F7* gene, it would be worth additionally testing for FX deficiency.

We identified 53 genetic variants initially classified as VUSs. After re-classification, 31 variants remained VUSs for which the pathogenicity prediction programs (MaxEntScan, NNSPLICE, SIFT and PolyPhen-2) showed inconsistence in pathogenicity estimation and put into question the reliability of these methods. Conformational data from in silico and in vitro analysis and evaluations of variant frequencies from larger population data sets or families are needed to confirm the role of these lesions on the function of the FVII protein.

We identified an underlying genetic defect in 390 IPs estimating a VDR of 55%, which linearly declines with the increase in the FVII:C and zygosity of the genetic defects. Compared to the VDR analyses of other deficiencies by Caspers et al., the estimated VDR for FVII deficiency was found close to that for protein S deficiency [23]. While in patients with FVII:C<10 IU/dl all genetic lesions were in homozygous or compound heterozygous states, with the increase in FVII:C, the proportion of the heterozygous presentation of defects expands and reaches nearly 100% in mild FVII deficiencies.

An interesting group comprised patients with FVII:C levels between 21 and 70 IU/dl, in whom no underlying genetic variant was detected (309 IPs). One possible explanation for this observation may be linked to recently described deep intronic variants in the *F7* gene, which are not routinely tested, or other genetic modifiers outside the *F7* gene [13]. Additionally, the accuracy of FVII:C assays, related to the sensitivity of the thromboplastin reagent of different sources [24] and the quality of the FVII-deficient plasma and calibrators used, may explain some inconsistencies in patients with relatively low FVII:C levels (11–20 IU/dl) where no genetic defect was identified, potentially leading to misdiagnosis of patients [15]. It is important to note that a limitation of this study is that FVII:C was not centrally tested, and we rely on the data reported by local labs. The availability of FVII:Ag levels and more standardized test methods such as the chromogenic FVII assay [15] could improve the diagnosis of FVII deficiency. Moreover, an acquired FVII deficiency, the concurrent presence of genetic defects in pro- and anti-coagulation factors, age and blood group [25] could not be ruled out.

The *F7* gene is also known to harbor several common variants that exert a significant influence on FVII:C. The most reported one is the missense variant c.1238G>A p.(Arg413Gln) (M1/M2 allele), associated with a 25% reduction in FVII:C [11]. The diverse distribution of this variant in the analyzed cohorts in this study suggests that both a pathogenic variant and the M2 allele can serve as alternative modulators of FVII protein synthesis and function. Our data showed that only 10% of individuals in the control group carried the M2 allele, and in the majority of cases, it was in a heterozygous state with M1, aligning with the frequency of 0.1, similar to what has been reported for the general European population [12]. In patients with severe and moderate deficiencies and an underlying genetic defect affecting both alleles, the M2 allele was predominantly in a heterozygous state. It can be speculated that in these cases the M2 variant might not significantly contribute to the reduction in FVII:C, but rather intensify the impact of co-inherited variants. The frequencies of the FVII:C-lowering M2 alleles were considerably high in patients with mild deficiencies and even higher in those without a pathogenic variant (69%) compared to those with a disease-causing variant (46%). This strongly indicates the association of M2 alleles with decreased FVII:C levels. In cases where FVII:C levels exceed 50 IU/dl, the homozygous status of the M2 allele might not lead to a clinical condition but rather mimic mild FVII deficiency. In line with the findings reported by Bernardi et al. [11], these results highlight the significance of assessing this genetic variant in the context of better interpretation of mild FVII deficiency.

Based on our observations, genetic analysis plays an increasingly important role in predicting and improving the diagnostic process of inherited FVII deficiency, especially in cases in which genetic counselling is needed. Moreover, the co-inheritance of common variants affects FVII:C and further complicates the diagnosis; thus, data interpretation should include the M2 variant. Accurate genetic diagnosis, particularly in mild and asymptomatic forms, is of importance, especially in preoperative conditions where the patients could be at risk of prolonged bleeding.

## 4. Materials and Methods

### 4.1. Patient and Control Cohorts

Between 2013 and 2020, our laboratory received 904 blood samples from individuals diagnosed with FVII deficiency for genetic analysis. The diagnosis of the FVII deficiency was assigned based on FVII:C levels (<70 IU/dl) though a local laboratory. Sixty-three index patients (IPs) were excluded due to the following criteria: a diagnosis of associated liver disease, inconsistent laboratory data or incomplete documentation. Furthermore, 137 family members were excluded, resulting in a final cohort of 704 IPs. The mean age of patients was 25 years, ranging from 1 to 86 years. Males comprised 54% (378 IPs) and females 46% (326 IPs) of the cohort. The control cohort consisted of 217 individuals without FVII deficiency (FVII:C > 70 IU/dl) and no pathogenic, likely pathogenic or variants of uncertain significance (VUSs) in the *F7* gene. The study was approved by the ethics committee of Bonn University of Medical Sciences (approval number 183/07).

### 4.2. Molecular Genetics Analyses

Genetic analyses were performed in the Department of Molecular Hemostaseology, University Hospital Bonn. Informed consent for molecular genetic analysis was obtained from each patient. Genomic DNA was isolated from peripheral EDTA blood using a Blood Core Kit (Qiagen, Hilden, Germany).

The *F7* gene (NM_000131) analyses were carried out on an ABI Prism 3130 genetic analyzer for Sanger sequencing (Thermo Fisher Scientific, Langenselbold, Germany) and a Mini-Seq genome sequencer (Illumina, Santa Clara, CA, USA) was used for next-generation sequencing (NGS). Data were evaluated by SeqScape Version 2.7 (Thermo Fisher Scientific) and SeqPilot (JSI Medical Systems, Ettenheim, Germany) software version 5.2. For the description of sequence variations at the DNA and protein level, the guidelines of the Human Genome Variation Society (HGVS https://varnomen.hgvs.org/ accessed on 1 July 2021) [26,27] were applied and variants were filtered according to minor allele frequency (MAF < 1% in gnomAD).

Large deletions and duplications were analyzed with multiplex ligation-dependent probe amplification (MLPA) analysis or copy number variation (CNV) analysis. MLPA was performed according to the manufacturer’s recommendations, using SALSA MLPA Kits (MRC-Holland, Amsterdam, The Netherlands). Dosage analyses were performed by Coffalyser (V5.2) software (MRC-Holland). CNV evaluation was achieved by SeqPilot (JSI medical systems GmbH, Ettenheim, Germany).

The genetic variant classification and criteria used for assessment variant pathogenicity was performed according to ACMG (American College of Medical Genetics, Bethesda, MD, USA) and AMP (Association for Molecular Pathology, Rockville, MD, USA) guidelines for the interpretation of sequence variants [28]. The disease causality of all genetic alterations was compared to the Human Gene Mutation Database (HGMD) [29], the ClinVar database [30] and the EAHAD database [7]).

## Figures and Tables

**Table 1:** Genetic variants that were identified in the cohort of 704 IPs that were not reported in the FVII deficiency databases.

Nr.	Exon/Intron	HGVS (Nucleotide Position)	HGVS (Protein Position)	Type of Variant	Classification	FVII:C (IU/dl)
1	Promotor	c.-4C>A	-	regulatory	VUS	31
2	1	c.44T>C	p.Leu15Pro **^++^**	missense	likely pathogenic	37
3	1	**c.48_64dup17ins**	**p.Gly17Alafs**	dup/ins	likely pathogenic	38
4	1	c.65G>A	p.Gly22Asp	missense	VUS	43
5	3	c.143A>T	p.Gln48Leu **^+^**	missense	likely pathogenic	20
6	3	c.218T>C **^#^**	p.Leu73Pro	missense	VUS	1
7	3	**c.220dupG**	**p.Glu74Glyfs**	duplication	likely pathogenic	35
8	3	**c.281_291del10bp**	**p.Ala94Glyfs**	deletion	likely pathogenic	40
9	4	**c.316+1G>A**	**-**	splice-site	likely pathogenic	43
10	5	c.343T>G	p.Cys115Gly **^+^**	missense	likely pathogenic	30
11	5	c.408C>G	p.Phe136Leu	missense	VUS	33
12	5	c.412G>C	p.Gly138Arg	missense	VUS	35
13	6	c.430C>G	p.His144Asp	missense	VUS	43
14	6	c.512C>T **^§^**	p.Ser171Phe **^++^**	missense	likely pathogenic	1
15	6	c.514T>C	p.Cys172Arg	missense	VUS	32
16	6	c.526G>A	p.Glu176Lys	missense	VUS	48
17	6	**c.538_539delCT**	**p.Leu180Alafs**	deletion	likely pathogenic	54
18	6	c.557C>T	p.Ser186Phe	missense	VUS	29
19	6	c.565C>T	p.Pro189Ser **^++++^**	missense	likely pathogenic	50
20	6	c.548A>T	p.Asp183Val	missense	VUS	28
21	7	c.587G>C	p.Gly196Ala	missense	VUS	33
22	7	c.665G>A	p.Gly222Glu	missense	VUS	30
23	7	**c.667G>T**	**p.Glu223Ter**	nonsense	likely pathogenic	57
24	7	c.676T>G	p.Trp226Gly	missense	VUS	36
25	7	**c.681+1G>T**	**-**	splice-site	likely pathogenic	52
26	8	**c.691_693delTTG ^#^**	**p.Leu231del**	deletion	likely pathogenic	8
27	8	c.718G>T	p.Gly240Trp **^+^**	missense	likely pathogenic	30
28	8	c.728T>C	p.Ile243Thr	missense	VUS	29
29	8	c.739T>C	p.Trp247Arg	missense	VUS	26
30	8	**c.806-3C>G**	-	splice-site	likely pathogenic	54
31	9	c.808G>A	p.Glu270Lys	missense	VUS	48
32	9	c.823G>A	p.Glu275Lys	missense	VUS	41
33	9	c.843G>T **^#^**	p.Gln281His	missense	VUS	1
34	9	c.903C>G	p.His301Gln **^+++^**	missense	likely pathogenic	59
35	9	c.904G>A	p.Asp302Asn	missense	VUS	30
36	9	c.911C>T	p.Ala304Val	missense	VUS	30
37	9	c.930G>T	p.Gln310His **^++^**	missense	likely pathogenic	43
38	9	c.944C>T	p.Thr315Ile **^+++^**	missense	likely pathogenic	19
39	9	c.955G>A	p.Val319Met	missense	VUS	50
40	9	c.977G>A	p.Arg326Gln	missense	VUS	37
41	9	c.1160T>C	p.Met387hr	missense	VUS	42
42	9	c.1168G>A	p.Ala390Thr	missense	VUS	43
43	9	c.1078C>T	p.Leu360Phe	missense	VUS	64
44	9	**c.1089_1090delCC**	**p. Arg364Alafs**	deletion	likely pathogenic	19
45	9	c.1235A>G	p.Tyr412Cys **^++^**	missense	likely pathogenic	43
46	9	c.1250A>G	p.Tyr417Cys	missense	VUS	45
47	9	c.1274G>C	p.Gly425Ala	missense	VUS	40
48	9	c.1262T>C	p.Ile421Thr	missense	VUS	45
49	9	c.1306G>A	p.Val436Met	missense	VUS	47
50	9	c.1313C>A **^#^**	p.Thr438Asn	missense	VUS	8
51	9	c.1316G>C **^#^**	p.Arg439Thr	missense	VUS	2
52	9	**c.1329C>G**	**p.Tyr443Ter**	nonsense	likely pathogenic	14
53	9	c.1354C>T	p.Arg452Cys	missense	VUS	37

VUS: variant of uncertain significance; HGVS: Human Genome Variation Society. ^#^: variants identified in a compound heterozygous state. ^§^: variants identified in a homozygous state. FVII:C: FVII activity. Variants reclassified as likely pathogenic due to the type are in bold; ^+^: variants reclassified as likely pathogenic due to family segregation analysis. Each ^+^ represents one family member carrying the same variant and reduced FVII:C levels. ^+^: 1 family member, ^++^ two family members, ^+++^: three family members, ^++++^: four family members.

## Data Availability

Data are contained within the article or Supplementary Materials.

## Supplementary Materials

The following supporting information can be downloaded at: https://www.mdpi.com/article/10.3390/ijms25042384/s1.
